# GNG Motifs Can Replace a GGG Stretch during G-Quadruplex Formation in a Context Dependent Manner

**DOI:** 10.1371/journal.pone.0158794

**Published:** 2016-07-14

**Authors:** Kohal Das, Mrinal Srivastava, Sathees C. Raghavan

**Affiliations:** Department of Biochemistry, Indian Institute of Science, Bangalore, 560012, India; Michigan State University, UNITED STATES

## Abstract

G-quadruplexes are one of the most commonly studied non-B DNA structures. Generally, these structures are formed using a minimum of 4, three guanine tracts, with connecting loops ranging from one to seven. Recent studies have reported deviation from this general convention. One such deviation is the involvement of bulges in the guanine tracts. In this study, guanines along with bulges, also referred to as GNG motifs have been extensively studied using recently reported *HOX11* breakpoint fragile region I as a model template. By strategic mutagenesis approach we show that the contribution from continuous G-tracts may be dispensible during G-quadruplex formation when such motifs are flanked by GNGs. Importantly, the positioning and number of GNG/GNGNG can also influence the formation of G-quadruplexes. Further, we assessed three genomic regions from *HIF1* alpha, *VEGF* and *SHOX* gene for G-quadruplex formation using GNG motifs. We show that *HIF1* alpha sequence harbouring GNG motifs can fold into intramolecular G-quadruplex. In contrast, GNG motifs in mutant *VEGF* sequence could not participate in structure formation, suggesting that the usage of GNG is context dependent. Importantly, we show that when two continuous stretches of guanines are flanked by two independent GNG motifs in a naturally occurring sequence (*SHOX*), it can fold into an intramolecular G-quadruplex. Finally, we show the specific binding of G-quadruplex binding protein, Nucleolin and G-quadruplex antibody, BG4 to *SHOX* G-quadruplex. Overall, our study provides novel insights into the role of GNG motifs in G-quadruplex structure formation which may have both physiological and pathological implications.

## Introduction

B-DNA is formed between two strands of the DNA in an antiparallel fashion by well-known Watson-Crick base pairing [1,2]. Over past few decades, there have been increasing evidences of DNA structures that deviate from the conventional B-DNA, often termed as alternative DNA structures or non-B DNA. G-quadruplex, cruciform, triplex, Z-DNA, etc. are some of the most common well studied non-B DNA structures [3–6]. Biophysical, NMR and crystallographic studies by several groups have provided useful insights into the structural details of these forms. Mostly, such altered DNA structures are formed at specific sequence motifs and can function as molecular platforms where proteins may bind [7]. All of these structures are very well characterised *in vitro* and their physiological roles have been either established or implicated *in vivo* (6).

In 1962, Gellert and colleagues demonstrated that guanylic acid can form remarkably stable tetra-molecular structures (quartets) [8]. These quartets have four guanines, each of which is paired to two of its adjacent guanines by hydrogen bonds in a square planar arrangement. Stacks of quartets result in the formation of G4 structure, wherein loops of 1–7 nucleotides connecting the G stretches are extruded [9]. The G4 structure generates an electronegative cage which can harbour monovalent cations, preferably K^+^, which has been shown to stabilize the structure. Despite the sequence bias, G-quadruplex structures are highly heterogeneous in nature. Monomeric, dimeric and higher order structures can be formed simultaneously by a single oligonucleotide under appropriate conditions. Small changes in sequence and experimental conditions can result in drastic changes in the folding pattern of G-quadruplexes [10]. Depending upon the strand polarities, G-quadruplexes can be broadly classified as parallel or anti-parallel, both of which exhibit characteristic circular dichroism spectra [11,12]. Based on the location of the loops that connect the G-strands, G-quadruplex structures can further be categorized into propeller, lateral and diagonal [13].

In the human genome, over 375,000 putative G4 forming motifs have been suggested to exist through computational analyses [14]. Although scepticism towards their *in vivo* relevance exists since discovery, growing *in vitro* and *in vivo* evidences have drawn interest and strengthened the conviction among scientists. G4 DNA has been evidenced in telomeric regions where such higher order structures together with definite set of protein complexes protect the chromosomal ends [15]. These structures play a very important role in gene regulation, acting as transcription regulators or roadblocks at promoter regions of several oncogenes [16,17]. Besides, G-quadruplexes can stall replication forks which may result in genomic instability [18]. Pathologically, G-quadruplexes are well known for causing genomic fragility which can result in chromosomal translocations [19–23]. Recently, RNA G-quadruplexes have also been shown to be physiologically relevant and have been implicated to play an important role in translation regulation and mRNA processing [24]. G-quadruplex structures have also gained importance in therapeutics. Multiple ligands that bind to these structures have been generated and some of them have shown remarkable anti-cancer properties [25–27].

Most of the G-quadruplex forming sequences follow an empirical formulae G_≥3_X G_≥3_X G_≥3_X G_≥3_, where the loop length (X) ranges from one to seven. Through NMR studies, Phan’s group showed that G stretches can have interruptions, and larger loop lengths can be accommodated, provided they fold into duplex hairpins [28,29]. Here, we investigated whether GNG motifs adjacent to G stretches could fold into G-quadruplex structures. Systematically and rationally, a series of oligonucleotides were designed to evaluate the role of GNGs during G-quadruplex formation at *HOX11* translocation breakpoint regions, which was used as a model system. We found that GNG motifs could be well accommodated within G-quadruplex structure. To validate the observation further, we assessed three other genomic regions harbouring GNG motifs viz. *HIF1* alpha promoter, *VEGF* promoter and *SHOX* gene. Interestingly, our results show that GNG involvement in structure formation is entirely context dependent. The nature and distribution of the guanine stretches greatly affect the GNG involvement. The number of GNG motifs can also have an impact on structure formation. Finally, we also show that both G-quadruplex binding proteins, Nucleolin and engineered antibody, BG4 can specifically bind to G-quadruplexes involving GNG sequences.

## Materials and Methods

### Enzymes, chemicals, and reagents

Chemical reagents were obtained from Sigma Chemical Co. (United States) and SRL (India). DNA-modifying enzymes were from New England BioLabs (United States) and Fermentas (United States). Radioisotope-labeled nucleotides were from BRIT (India).

### Oligomers

The DNA oligonucleotides used in this study are enlisted in S1 Table. Oligomers were purified using 10–15% denaturing polyacrylamide gel electrophoresis followed by phenol-chloroform extraction and precipitation and dissolved in TE (10 mM Tris, 1 mM EDTA, pH 8.0) buffer [30].

Oligonucleotides spanning *HOX11* translocation breakpoint region I (MN38 and its reverse complement MN37) were designed and commercially synthesized. Mutant oligomers were generated by increasing the variability in DNA sequence step wise. In each case, first any of the four G stretches of MN38 was changed and keeping that as a backbone, other mutations were introduced. The mutant oligomers for *VEGF* and *HIF1-alpha* were also generated similarly (S1 Table).

### 5’-end labeling

The purified oligonucleotides were resuspended in TE (pH 8.0) and used for radiolabeling. The 5’ end of the oligonucleotides was labeled using T4 polynucleotide kinase in a buffer containing 20 mM Tris-acetate (pH 7.9), 10 mM magnesium acetate, 50 mM potassium acetate, 1 mM DTT and [γ-^32^P] ATP at 37°C for 1 h. The labeled substrates were purified using Sephadex G25 column and stored at -20°C until further use [31].

### Electrophoretic gel mobility shift assay (EMSA)

#### DNA EMSA

The radiolabeled oligomers were incubated either in the presence or absence of 100 mM KCl (indicated lane specific manner in each EMSA gel) in Tris–EDTA (TE) buffer (pH 8.0) at 37°C for 1 h. The different forms of G-quadruplexes were then resolved on 15% native polyacrylamide gels in the presence or absence of 100 mM KCl (indicated as +KCl or–KCl at the bottom of all EMSA gels), both, in the gel and the buffer, at 150 V at room temperature. TBE (1x) was used as running buffer in all cases. The gels were dried and exposed to a PhosphorImager cassette, and the signal was detected using PhosphorImager FLA9000 (Fuji, Japan). In one of the experiments, sample preparation was also carried out following heat denaturation of the oligonucleotides at 95°C (10 min) and gradually cooling at 37°C (O/N). EMSA with increasing concentration of labeled oligomers viz. MN38 and MS113 was carried out in a similar way, wherein the reactions were devoid of KCl, instead gel and buffer contained 100 mM KCl. The oligomer concentrations 0.5, 1, 1.5, 2, 3 and 4 nM were chosen for the studies.

#### Protein EMSA

Radiolabeled oligomers were incubated in presence of 100 mM KCl in binding buffer at 37°C for 1 h. For recombinant His-tagged Nucleolin, 100 ng of protein was added and the reaction was incubated at 4°C for 2 h. The bound complexes were then resolved on 8% native polyacrylamide gels in the presence of 100 mM KCl, added in the gel and the buffer, at 100V at 4°C. For, BG4, 2 μl of the control and immunoprecipitation (IP) reaction volumes each, were incubated with the oligonucleotide and the reaction was incubated at 37°C for 30 min. The bound complexes were then resolved on 6% native polyacrylamide gels in the presence of 100 mM KCl, added in the gel and the buffer, at 50 V at RT. The gels were dried and exposed to a screen, and the signal was detected using PhosphorImager FLA9000 (Fuji, Japan).

### Circular dichroism (CD)

Gel purified oligomers were incubated either in the presence or absence of 100 mM KCl, in TE at 37°C for 1 h. Circular dichroism spectra were recorded at RT from 200 to 300 nm and 5 cycles were accumulated for every sample, using a JASCO J-810 spectropolarimeter at a scan speed of 50 nm/min. Separate spectrum was measured for buffer alone for 5 cycles and this was subtracted from all the experimental spectra. The ellipticity was calculated using the software, Spectra Manager, and plotted as a function of wavelength [32,33]. For binding studies with Nucleolin and BG4, appropriate oligomers were incubated in presence of 100 mM KCl, in 1XPBS at 37°C for 1 h. In case of Nucleolin, 20 μg of protein was incubated with the oligonucleotide at 4°C for 30 min and the spectrum was recorded at 4°C. In case of BG4, 5 μg of protein was incubated with the oligonucleotide at 37°C for 30 min and the spectrum was recorded at 37°C. Other measurement parameters for protein binding were kept constant.

### Purification of intramolecular GNG G-quadruplex

Purification of intramolecular GNG G-quadruplex was carried out for MS113. 12 μl of MS113 (146.2 μM) was used for EMSA. Labeled MS113 and MN37 were used as reference. All the three oligonucleotides were heat denatured and gradually cooled to 37°C and then loaded onto a 15% native PAGE in presence of KCl and electrophoresed (12 h at 60 V). The gel was exposed to a phosphorImager screen, and the signal was detected using PhosphorImager FLA9000 (Fuji, Japan). Based on position of intramolecular species, the corresponding band of cold MS113 was excised from an adjacent lane from the gel. The intramolecular species were then eluted from the gel and CD studies were carried out as mentioned earlier.

### DMS (Dimethyl sulfate) protection assay

DMS protection assay was carried out on radiolabeled KD52 (harbouring two G stretches and three GNG motifs) as described previously with modification [19,22]. KD52 was incubated in TE or in presence of either 100 mM KCl or LiCl dissolved in TE, at 37°C for 1 h. Dimethyl sulfate (DMS) was added to the reaction mixture (1/200 dilution) and incubated for 15 min at RT. Equal volume of piperidine (10%) was added to each reaction and incubated at 90°C for 30 min. Equal volume of water was added to the reaction mixture and vacuum dried; the pellet formed was further washed with water thrice and dried using a speedvac concentrator. The reaction products were resolved on a 15% denaturing polyacrylamide gel, dried and visualized as described above.

### Overexpression and purification of Nucleolin

A recombinant pET28a plasmid carrying a truncated nucleolin gene encoding amino acid residues 284–710 (ExPASy predicted molecular weight is 48 kDa, but the SDS PAGE profile showed Nucleolin band at ~55 kDa, whose identity was confirmed through western blotting) was generated, pNu. *Escherichia coli* Rosetta cells transformed with pNu were grown till *A*_600_−0.8 and then induced with 0.6 mM IPTG at 37°C for 3 h as described previously with modification [34]. After induction, cell pellets were re-suspended in protein extraction buffer (50 mM sodium phosphate, 300 mM NaCl, 1 mM PMSF and 1 mM DTT) and lysed by sonication. The lysate was centrifuged at 14,000 rpm at 4°C for 15 min. The supernatant was loaded onto a Ni-NTA column and the bound protein was eluted using increasing concentration of Imidazole (100 to 400 mM). The protein was further dialysed in buffer containing 20 mM Tris-HCl, 150 mM NaCl, 10% Glycerol, 1 mM DTT; at 4°C for 16 h. Protein purity and identity was assessed by SDS-PAGE and western blotting.

### Purification and immunoprecipitation using anti-BG4

The plasmid expressing BG4 protein, pSANG10-3F-BG4 was a gift from Shankar Balasubramanian (Addgene plasmid # 55756). The plasmid was transformed into BL21(DE3) bacteria, and the transformed bacteria were grown at 30°C, upto an O.D. of 0.6. Induction was carried out with 1 mM IPTG for a period of 16 h at 16°C, harvested, and resuspended in lysis buffer (20 mM Tris-HCl [pH 8.0], 50 mM NaCl, 5% glycerol, 1% Triton X-100 and 1 mM PMSF). The cells were lysed by sonication, centrifuged, and the supernatant was then loaded onto a Ni-NTA chromatography column (Novagen). BG4 was eluted using increasing concentrations of Imidazole (100–300 mM). Dialysed BG4 fractions were used for IP and binding assay. For IP 3.6 μg of BG4 (4 μl of 900 ng/ μl) was incubated with 3 μl of anti-FLAG antibody in 1X PBS at 4°C for 16 h. Then 7 μl of Protein G agarose beads (Sigma, St. Louis, MO, USA) was added to the reaction mixture (total reaction volume 40 μl) and incubated at 4°C for 16 h. The beads were spun down at 1500 rpm for 1 min and the supernatant was separated and used for the assay. A control reaction was carried out following the same procedure without the addition of anti-FLAG. Immunodepletion was analysed through western blot relative to the control reaction.

## Results

### G-quadruplex structure formation at HOX11 translocation breakpoint region is dependent on GNG motifs, when guanine stretch is mutated

**Fig 1:**
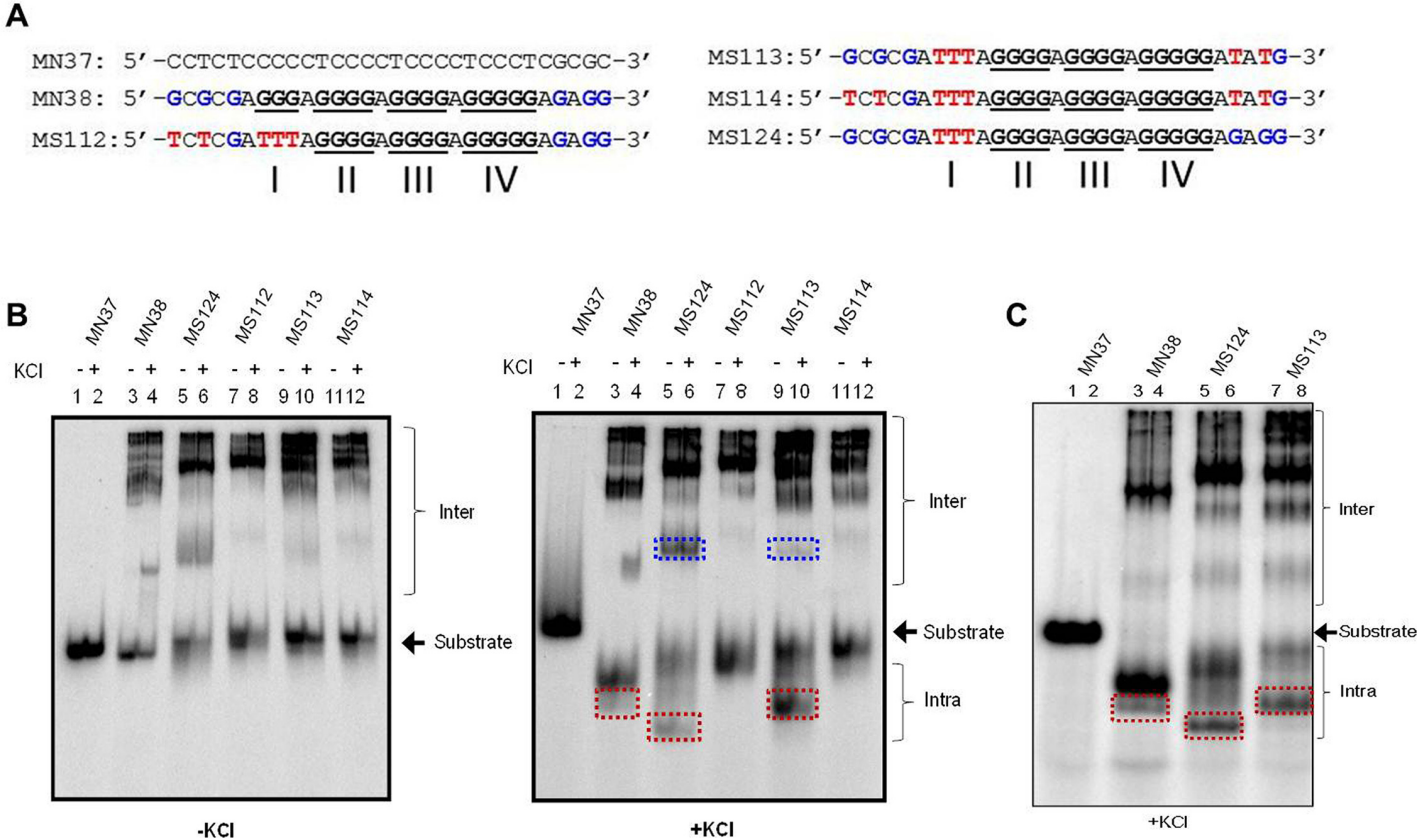
Evaluation of effect of GNG motifs in G-quadruplex structure formation at HOX11 translocation breakpoint region. (A) The oligomeric DNA sequence spanning region I of chromosomal translocation breakpoint region from *HOX11* gene. A G-rich strand (MN38), its mutants (MS112, MS113, MS114, MS124) and complementary C rich strand (MN37) are designed based on sequence of *HOX11* breakpoint region and used for the study. The mutations were introduced such that the first G stretch is altered to thymines (indicated in red) and keeping that as the backbone, the first GNG motif (indicated in blue) or the second GNG motif (indicated in blue) or both are mutated (mutations shown in red). (B) Radiolabeled oligomeric DNA with G-rich, C-rich or mutant DNA sequence were incubated in the presence (+) or absence (-) of KCl (100 mM) and resolved in the absence (left panel) or presence (right panel) of KCl (100 mM) in the gel and running buffer. (C) Radiolabeled oligomeric DNA with G-rich, C-rich or mutant DNA sequence were heat denatured, gradually cooled and resolved in the presence of KCl in the gel and running buffer. For each oligonucleotide duplicate reactions are loaded in adjacent lanes. The substrate, intramolecular (Intra), and intermolecular (Inter) quadruplex structures are indicated. The red boxed intra indicate intramolecular G-quadruplex species involving GNG motifs. The blue coloured box indicate bimolecular G-quadruplex.

Previous studies have shown that t(10;14) chromosomal translocation breakpoint region from *HOX11* gene can fold into intra and inter molecular G-quadruplex structures [22]. In the present study, we assessed the importance of different stretches of guanines, in the context of GNG motifs during the formation of G-quadruplex at region I of *HOX11* breakpoints. Oligomeric DNA spanning the G-rich strand (MN38) and its complementary sequence (MN37) were designed and synthesized (Fig 1A). MN38 has four core guanine stretches (I, II, III and IV) and two GNG motifs flanking the core sequence (Fig 1A). Both, the upstream GNG (1^st^ GNGNG) and the downstream GNG (2^nd^ GNGG) motifs contain three guanines wherein all guanines in the 1^st^ GNG motif are spaced by one nucleotide and two 5’ guanines of the 2^nd^ GNG motif are spaced by an adenine (Fig 1A). Conventionally, the core sequence of MN38 is sufficient for formation of G-quadruplex. We wondered whether G-quadruplex structure formation involves any of the available GNG motifs. In order to test this, we have designed oligonucleotides in which guanine stretch I was mutated to thymines (MS124). Besides, oligomers were also designed using MS124 as backbone in which 1^st^ GNG, 2^nd^ GNG or both (MS112, MS113 and MS114, respectively) were mutated (Fig 1A). EMSA was performed in presence and absence of KCl after radiolabeling the oligonucleotides (Fig 1B). C-rich oligomer, MN37 served as a negative control. Since C-rich DNA is known not to form structure in neutral pH, it also acts as a molecular weight marker for G-rich oligomer of same length. If the G-rich DNA runs with similar mobility as the C-rich DNA, then it can be considered not to form any structure. Faster mobility of the G-rich DNA with respect to C-rich strand is regarded as intramolecular G-quadruplex formation and slower mobility of G-rich DNA is regarded as intermolecular G-quadruplex formation as reported previously [19,22].

Results showed varying degrees of higher order structure formation irrespective of the presence of KCl (Fig 1B). The folding and nature of G-quadruplex is characteristic to each oligonucleotide sequence and % formation of intermolecular or intramolecular species also depends on the same. Moreover, the differential mobility shift for each oligonucleotide, observed in EMSA depended completely on the respective structures formed. Although, majority of the DNA molecules in our study formed intermolecular G-quadruplex, here we mainly focus on formation of intramolecular species utilizing noncanonical sequences or GNGs. Intramolecular G-quadruplex formation was undetectable for all the oligonucleotides, when EMSA was performed without KCl. Interestingly, addition of KCl in gel and buffer resulted in intramolecular G-quadruplex in wild type oligomer MN38, and mutants MS124 and MS113 (Fig 1B), which was absent in case of other mutants, MS112 and MS114 (Fig 1B). Formation of intramolecular G-quadruplex requires a minimum of four G stretches interspersed by loops. Thus, formation of intramolecular species in case of MS124 and MS113 is possible only if GNG motifs are involved during the structure formation. Owing to conformational variability of G-quadruplex structures, the intramolecular species formed in MS124 and MS113 have differential mobility, whereas the mobility of MS113 and MN38 is nearly same (Fig 1B, boxed). Our results suggest that in case of MS113, the 1^st^ GNG motif is involved in intramolecular G-quadruplex structure formation. Moreover, these intramolecular GNG G-quadruplex species were able to form even after complete denaturation followed by gradual cooling (Fig 1C). Additionally, a bimolecular G-quadruplex is also formed (Fig 1B, right panel, Lanes 5, 6, 9,10) only in case of MS124 and MS113 suggesting the involvement of 1^st^ GNG motif in the formation of intermolecular G-quadruplexes. However, we did not find a role for 2^nd^ GNG motif during the G-quadruplex formation.

**Fig 2:**
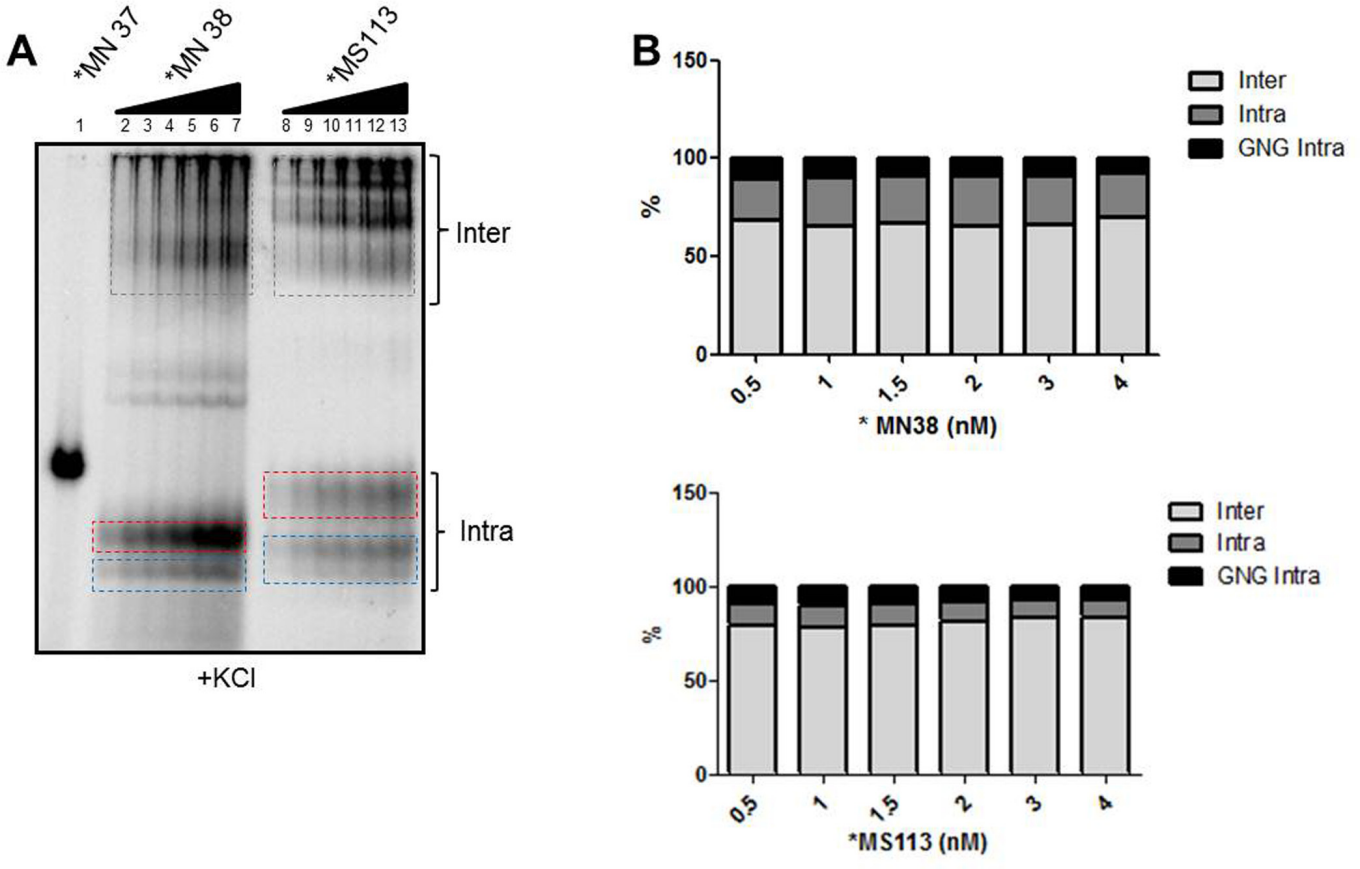
Impact of increase in concentration of oligomers on formation of inter and intramolecular G-quadruplex species. The wild type DNA sequence spanning region I of *HOX11* breakpoint region, MN38 (G-rich strand) or MS113 (mutant) was used for titration. MN37, the C-rich strand was used as a negative control. (A) EMSA gel shows formation of inter (boxed grey) and intramolecular G-quadruplexes. The intramolecular G-quadruplexes were further divided into species with (boxed blue) or without (boxed red) GNG motifs. (B) Bar diagram shows quantification of inter and intramolecular species over a range of oligonucleotide concentrations.

To evaluate whether the formation of GNG involving intramolecular G-quadruplexes was dependent on oligonucleotide concentration, we performed EMSA with varying concentration of MN38 and MS113. It was observed that the relative amount of intermolecular species (Fig 2, boxed grey), intramolecular species (Fig 2, boxed red) and GNG involving intramolecular species (Fig 2, boxed blue) remained constant over a range of concentrations. This suggests that folding of oligonucleotides into intramolecular species in presence or absence of GNG was not dependent on oligomer concentration.

Circular dichroism (CD) studies were carried out using the oligonucleotides in presence and absence of KCl. All the oligonucleotides showed a characteristic positive peak at 260 nm and a negative peak at 240 nm indicating formation of parallel G-quadruplex irrespective of the presence of KCl (S1 Fig). One limitation of this biophysical technique is that it cannot distinguish between intra and inter molecular G-quadruplex species. However, in this case majority of the oligonucleotides fold into higher order intermolecular structure, which contributes maximally to the observed spectra (S1 Fig).

### Involvement of GNG motif during G-quadruplex structure formation is context dependent

**Fig 3:**
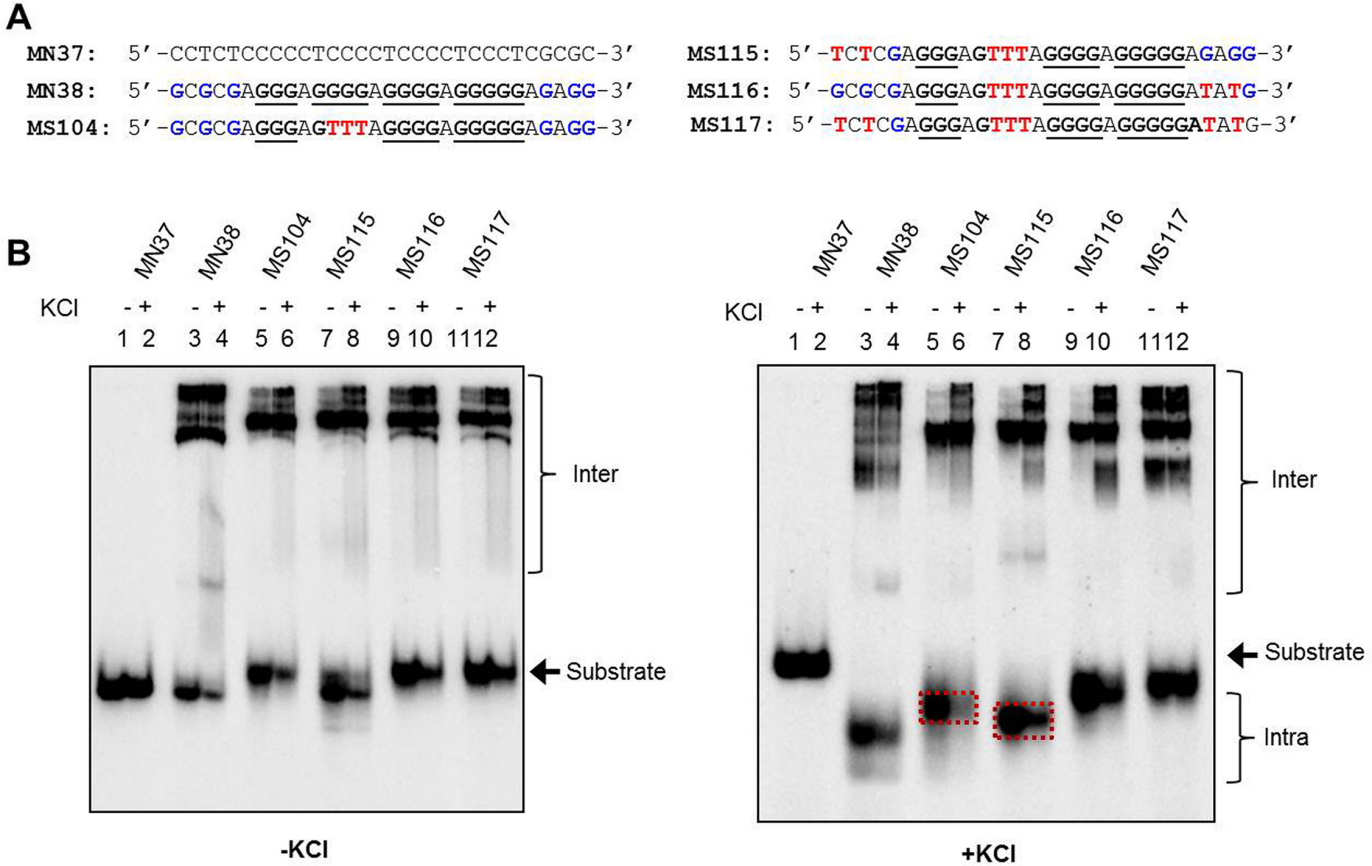
Analysis of involvement of GNG motif on G-quadruplex structure formation, when second stretch of guanines was mutated at *HOX11* breakpoint region I. (A) The oligomeric DNA sequence spanning region I, a G-rich strand (MN38) and complementary C rich strand (MN37) were designed from the *HOX11* breakpoint region. Mutants of MN38 were also designed and named as MS104, MS115, MS116, MS117. The mutations were introduced such that the second G stretch is altered to thymine (indicated in red) and keeping that as backbone either the first GNG motif (blue) or the second GNG motif (blue) or both are mutated (indicated in red). (B) The radiolabeled G- and C-rich strands along with the mutants were incubated in the presence (+) or absence (-) of KCl (100 mM) and resolved in the absence (left panel) or presence (right panel) of KCl (100 mM), in the gel and running buffer. The substrate, intramolecular (Intra), and intermolecular (Inter) quadruplex structures are indicated. The red boxed ‘Intra’ indicate intramolecular G-quadruplex species involving GNG motifs.

In the previous section we showed that when first stretch of guanines was mutated, G-quadruplex formation was dependent on 1^st^ GNG motif. We wondered whether same holds true, when second stretch guanine is mutated instead of first one. To evaluate this possibility, we have mutated the second G stretch to thymines (MS104) and then mutations were introduced within 1^st^ GNG (MS115), 2^nd^ GNG (MS116) or both (MS117) in the context of MS104 (Fig 3A). EMSA studies were carried out using all oligonucleotides in presence and absence of KCl. Interestingly, we observed that mutation at G stretch II completely changed the GNG usage pattern (Fig 3B, right panel). Unlike MS116 and MS117, both MS104 and MS115 formed intramolecular G-quadruplex structure as evident by the altered mobility in presence of KCl (Fig 3B, right panel, Lanes 5–8). This suggests the involvement of 2^nd^ GNG motif during G-quadruplex structure formation in case of MS104 and MS115, which is abrogated when the motif is mutated. Thus, the choice of GNG involvement varies depending on the context of G-stretches present. As seen before, although majority of the oligonucleotides fold into higher order structures, the percentage of inter molecular species formed in each case is higher in KCl containing reactions irrespective of the presence or absence of KCl in gel and running buffer.

### Positioning of G stretches could dictate usage of GNG motifs

Having studied GNG choice and its relevance with I and II stretches of guanines, we wondered if modification of other guanine stretches (III and IV) would have an impact on G-quadruplex structure formation and in the usage of GNG motifs. We designed oligonucleotides with mutations in G stretches III (KD40) and IV (KD41). Besides, 2^nd^ GNG motif of KD40 and KD41 was mutated generating KD38 and KD39, respectively (S2A Fig). Interestingly, we observed no intramolecular G-quadruplex formation in the case of KD40 and KD38 upon EMSA analysis (S2B Fig, right panel, lanes 5–8). ~90% of KD40 and KD38 were utilized during formation of higher order intermolecular structures. In contrast, KD41 showed weak intramolecular G-quadruplex formation in presence of KCl, which was absent otherwise. Disappearance of the intramolecular species upon mutation of 2^nd^ GNG (KD39) indicated its involvement in structure formation (S2B Fig). These results in conjunction with previous ones show that mutations in different stretches of guanines affect intramolecular G-quadruplex formation to varying degrees. More importantly, intramolecular G-quadruplex formation by these mutants involves GNG motif as an integral part of their structure, and usage of GNG is context dependent.

### All guanines within the GNGNG motif participate in structure formation when G stretch I is mutated

**Fig 4:**
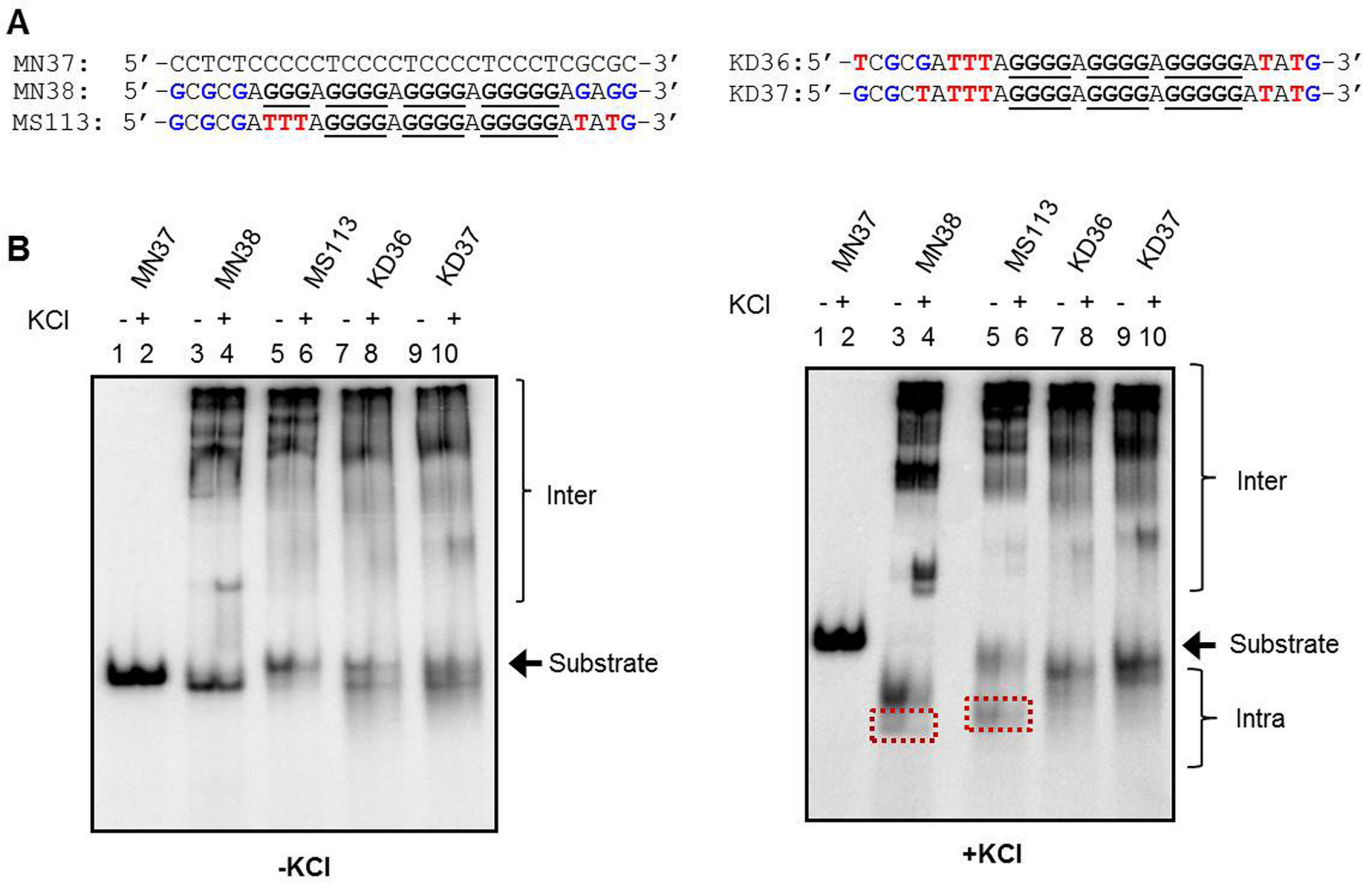
Evaluation of role of individual guanines in a GNGNG motif during G-quadruplex formation. (A) The oligomeric DNA sequence with mutations were made using MS113 as template wherein the guanines in the first GNG motifs were altered sequentially to thymine (indicated in red) generating KD36 and KD37. (B) The G- and C-rich strands along with the mutants were incubated in the presence (+) or absence (-) of KCl (100 mM) and resolved in the absence (left panel) or presence (right panel) of KCl (100 mM), in the gel and running buffer. The substrate, intramolecular (Intra) and intermolecular (Inter) quadruplex structures are indicated. The red boxed “intra” indicate intramolecular G-quadruplex species involving GNG motifs.

**Fig 5:**
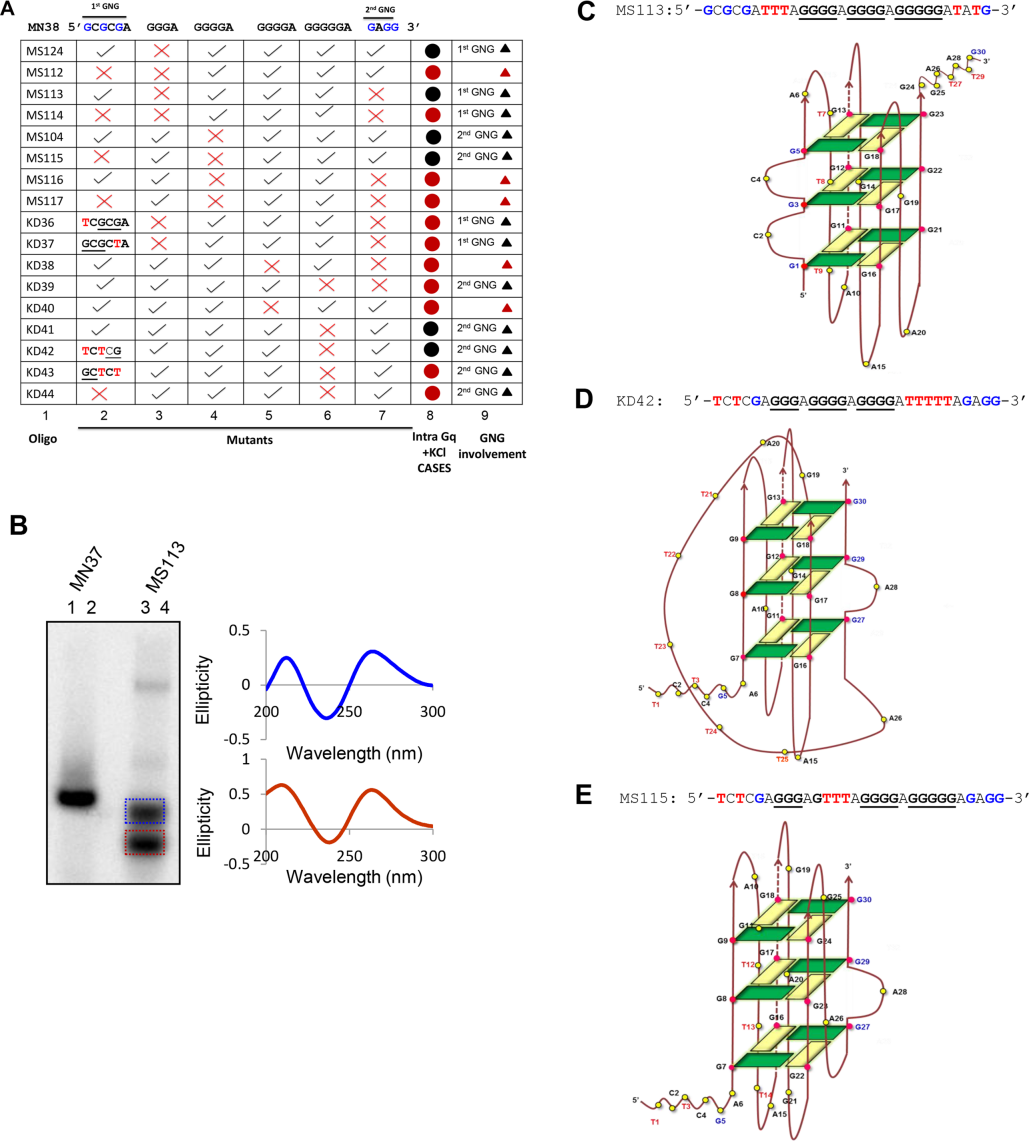
Summary of oligomeric DNA studied that support intramolecular G-quadruplex formation using GNG motifs and cartoon showing 2-D models of potential G-quadruplex structures. (A) Summary of oligomeric DNA of *HOX11* region I, and its mutants used in the study and their potential to fold into intramolecular G-quadruplex using GNG motifs. The red cross (from 2^nd^ to 7^th^ column) represents alterations in the DNA sequence and the black ‘tick mark’ represents no change in sequence. The red circle in the 8^th^ column indicates no formation of intramolecular G-qudraplex involving GNG motifs. The black circle in the 8^th^ column indicates formation of intramolecular G-quadruplex (Intra Gq) involving GNG motifs, under 100 mM KCl condition. The 9^th^ column provides an inference depending on the EMSA result, indicating whether GNG motifs are involved (black triangle) during intramolecular G-quadruplex structure formation and whether it is the 1^st^ GNG or the 2^nd^ GNG motif that is actually accommodated in the intramolecular structure. Black triangles for KD36, KD37, KD39, KD43 and KD44 suggest GNG involvement because their respective mutation abrogated intramolecular GNG motif involving G-quadruplex structure formation. The red triangle indicates no intramolecular G-quadruplex structure formation. (B) Left panel shows the mobility shift of MS113 intramolecular species in comparison to MN37 when resolved on a native PAGE containing KCl (100 mM). Unlabeled MS113 was loaded next to radioactive oligomer lane in the same gel and was electrophoresed. Based on the PI image, corresponding position of the two boxed bands (blue and red) in the unlabeled MS113 lane were excised out and intramolecular species were eluted subsequently. These two intramolecular species were then subjected to CD analysis (the spectrum and its colour correspond to the respective bands boxed in the gel). (C-E) 2-D model representation of G-quadruplexes involving GNG motifs based on the EMSA results. The pink circles indicate guanines involved in G-tetrad formation. 2-D model depicting intramolecular G-quadruplex formation on oligomers MS113 (C), KD42 (D) and MS115 (E) are shown.

Generally, a minimum of three guanines is needed in each of the four G stretches during formation of G-quadruplex structures. We tested whether participation of all guanines in a GNG motif is essential for intramolecular G-quadruplex formation. We chose MS113, which folds into intramolecular G-quadruplex involving 1^st^ GNG motif, and mutated either the 5’ or 3’ guanine of the 1^st^ GNG, generating KD36 and KD37, respectively (Fig 4A). Complete abrogation of the intramolecular G quartet species in case of KD36 and KD37 upon EMSA in presence of KCl suggests that participation of all guanines of 1^st^ GNG is critical during G-quadruplex structure formation (Fig 4B, right panel 7, 8, 9, 10). In a second case we mutated KD41, which involves 2^nd^ GNG motif in intra molecular structure formation, to KD42, KD43 and KD44 (Fig 5A). Since, G stretches I, II and III along with the 2^nd^ GNG are intact in KD41, KD42, KD43 and KD44, formation of intra molecular species was envisaged in all cases. However, we observed only weak (KD42; data not shown) or no intramolecular species formation (KD43 and KD44; Fig 5A). These results suggest that both, 1^st^ and 2^nd^ GNG can be involved in structure formation, however in a context specific manner.

### 2-D model of potential G-quadruplexes involving GNG motifs

Among the analyzed oligonucleotides, we chose MS113, MS115 and KD42 to build the 2D-model (Fig 5B–5E). The models were built to form a three plate G-quadruplex structure, as described previously [35]. For MS113, we gel purified intramolecular species and used for CD studies (Fig 5B). The CD spectrum depicts intramolecular structure with parallel strand orientation (Fig 5B). Since, G-triplet and G-quartet share similar stacking and loop geometry, the CD spectra representing the strand orientation in the G-quadruplex may also hold true for G-triplex [36]. However, it can be envisaged that a G-quadruplex will have higher mobility than a G-triplex formed by same sequence owing to its more compact structure. Thus, among the purified intramolecular species of MS113, one represents intramolecular GNG G-quadruplex (Fig 5B, red boxed); while second one may account for a G-triplex (Fig 5B, blue boxed).

Since the CD spectra suggested parallel G-quadruplex conformation for MS113, the models were built maintaining parallel orientation of the strands (Fig 5B, left panel, S3A and S3B Fig). The loops connecting the guanines in the models are not scaled. MS113 depicts the involvement of 1^st^ GNG in structure formation accommodating two consecutive bulges in the structure (Fig 5C). On the other hand KD42 and MS115 may include only 2^nd^ GNG motif (Fig 5D and 5E). Although, our data show involvement of GNG motifs in structure formation, the precise involvement of guanines in G-quadruplex structures, observed through gel mobility cannot be commented upon. As mentioned earlier, G-quadruplex structures can vary remarkably with small sequence changes resulting in conformational heterogeneity. Fig 5A provides a summary on involvement of GNG motifs of different mutants generated from *HOX11* region I in the formation of intramolecular G-quadruplex structures.

### Genomic sequences harboring GNG motifs can fold into G-quadruplexes, when present along with stretches of guanines

**Fig 6:**
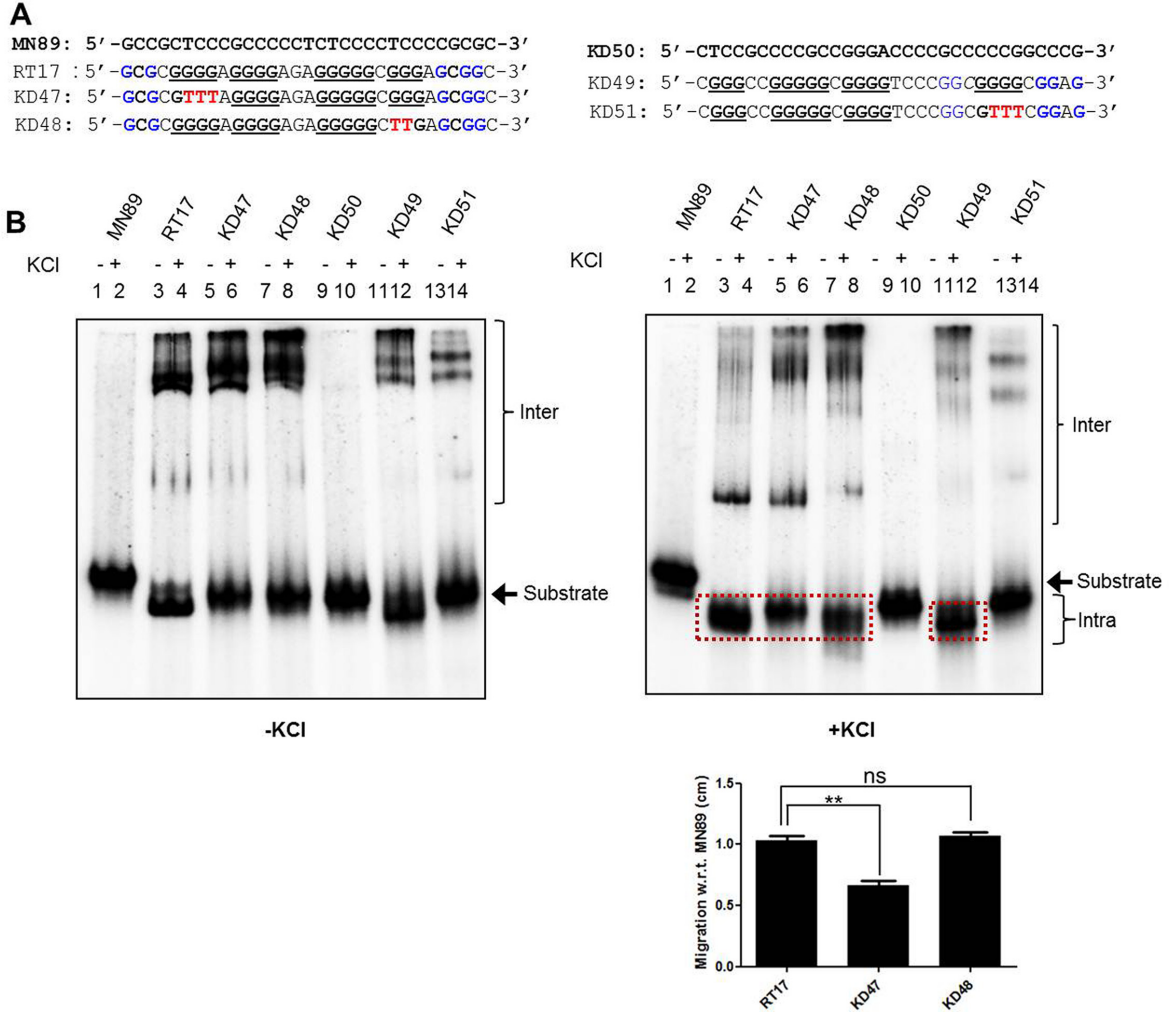
Evaluation of different genomic sequences harboring guanine stretches and GNG motifs for their ability to fold into G-quadruplex structures. (A) The oligomeric DNA spanning portion of *HIF1* alpha promoter and *VEGF* promoter. In both the cases, G-rich strand (RT17 and KD49 are from *HIF1* alpha and *VEGF*, respectively), its mutants (KD47 and KD48 are RT17 mutants; KD51 is the mutant of KD49) and complementary C-rich strand (MN89 and KD50 are reverse complement of RT17 and KD49, respectively) are depicted. In the case of *HIF1* alpha the mutations were made in the first and the fourth G stretch sequence, while it was the fourth G stretch in the case of *VEGF* sequence. (B) Native PAGE profile showing inter and intramolecular G-quadruplex formation at *HIF1* alpha promoter and *VEGF* promoter. The G- and C-rich strands along with the mutants were incubated in the presence (+) or absence (-) of KCl (100 mM) and resolved in the absence (left panel) or presence (right panel) of KCl (100 mM). For other details, refer Fig 1 legend. The bar diagram at the lower portion of the right panel represents relative migration of the boxed bands for RT17, KD47 and KD48 with respect to MN89.

It is known that putative G-quadruplex motifs are spread throughout the genome. Thus, the presence of GNGs associated with guanine stretches may widen the horizon of potential structure forming motifs *in vivo*. In this context we investigated the potential of other genomic sequences harboring GNG motifs to fold into structure. Several studies have previously shown that promoter regions of *HIF1*-alpha [19] and *VEGF* [9] can fold into G-quadruplex structure. Upon reexamination of these sequences we observed that along with the standard G stretches, they harbor flanking GNG motifs (Fig 6A). To assess the involvement of GNG in structure formation, we strategically mutated the G stretch I and IV of *HIF1* alpha and G stretch IV of *VEGF* (Fig 6A). The radiolabeled oligomers were resolved on native PAGE following incubation in KCl containing buffer as described above. Results showed that DNA sequences spanning 4 stretches of guanines from both *HIF1* alpha and *VEGF* were able to fold into intra and intermolecular G-quadruplex (Fig 6A and 6B), which was consistent with previous studies. Interestingly, KD48, mutant of *HIF1*-alpha showed robust intramolecular G-quadruplex formation, just like the wild type oligomer (RT17), indicating GNG involvement during the structure formation (Fig 6B, right panel, lanes 3, 4, 7, 8). However, the second mutant KD47 failed to do so (Fig 6B, right panel, lanes 3, 4, 5, 6), further reinstating the above observation that positioning of G-stretches play a critical role during GNG involvement and structure formation. Comparable result was observed when the samples were allowed to fold back after denaturation (S4B Fig) and the relative migration with respect to MN89 (C-rich control) is depicted in the graph (Fig 6B). In addition to that, KD51, a mutant of *VEGF* failed to fold into intramolecular species, further confirming that context of the GNG motif with respect to G-stretch is very critical when G-quadruplex structure is formed.

### Two guanine stretches when flanked with two independent GNG motifs in SHOX gene can fold into G-quadruplex structures

**Fig 7:**
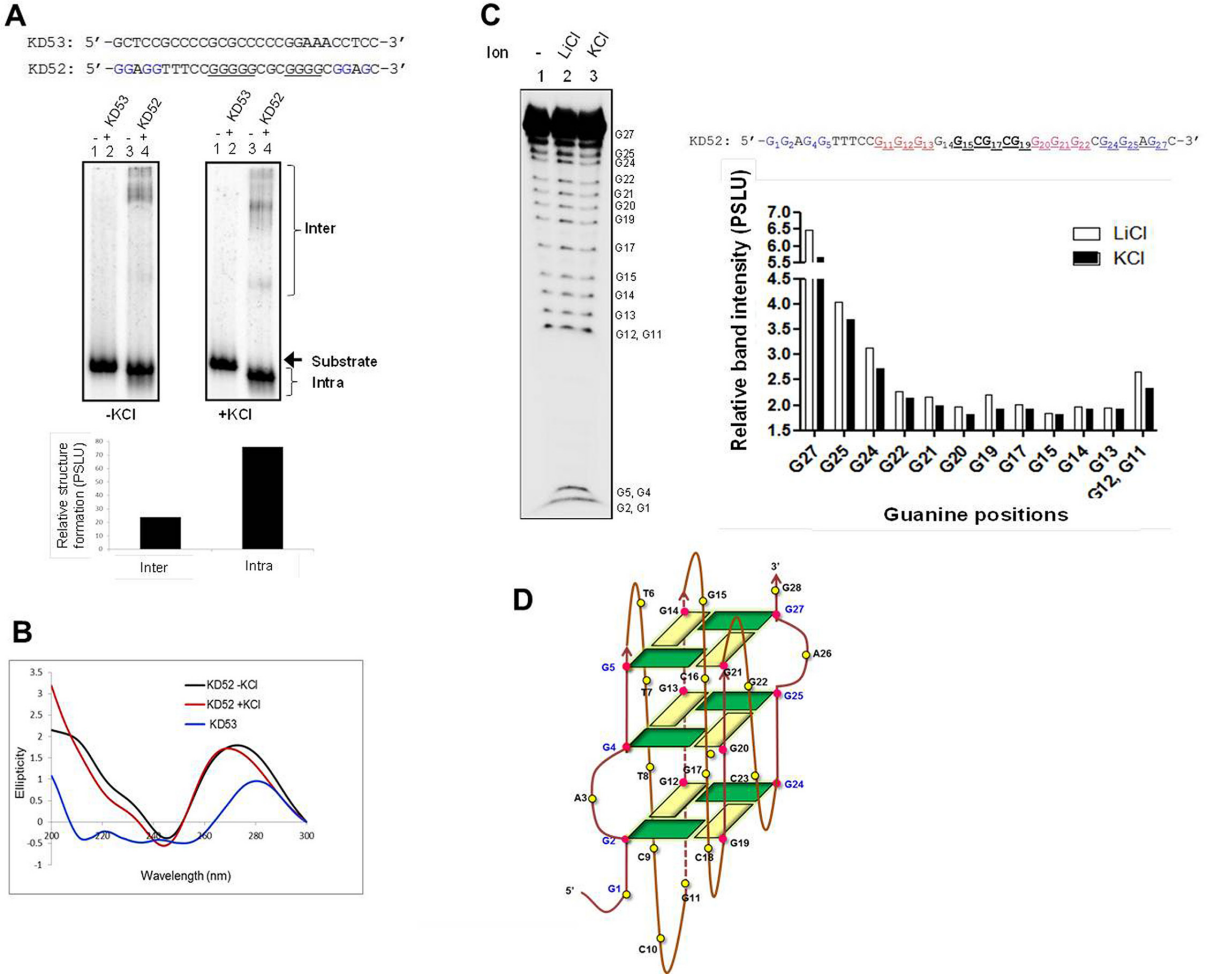
*SHOX* gene intramolecular G-quadruplex uses two GNG motifs for structure formation. (A) The oligomeric DNA sequence spanning portion of *SHOX* gene (KD52) and its complementary C rich strand (KD53). Native PAGE profile shows the formation of inter and intramolecular G-quadruplex in presence of KCl (100 mM). For other details, refer Fig 1 legend. Quantification for inter and intra G-quadruplex species are also shown. (B) CD analysis of KD52 and KD53 in presence of KCl (100 mM). (C) DMS assay on KD52. Radiolabelled KD52 was treated with DMS in presence of either KCl (100 mM) or LiCl (100 mM). Chemically modified DNA was then cleaved using piperidine and the reaction products were resolved on 15% denaturing PAGE (left panel). Quantification for individual guanine bands (in LiCl and KCl cases) is depicted in the right panel. Individual bands corresponding to each guanines were quantified from the lanes 2 and 3 using Multi Gauge and relative band intensities are plotted pairwise. (D) 2-D model showing potential intramolecular G-quadruplex formation on KD52.

Based on the above observations, we wondered whether a DNA sequence containing two G stretches flanked by a GNG motif, each on either side can fold into intramolecular G-quadruplex structures. To test this, we chose a natural sequence belonging to *SHOX* gene, KD52 (Fig 7A), which can fold into intramolecular species only if both the GNG motifs are involved. Results showed that KD52 can robustly fold into intramolecular G-quadruplex species in presence of 100 mM KCl (Fig 7A, lower right panel, lanes 3 and 4). Interestingly, on a KCl gel, ~75% of the DNA was in the intramolecular conformation (Fig 7A). Besides, renaturation of KD52, following denaturation yielded similar result (S4A Fig). CD analysis of KD52 also showed formation of parallel G-quadruplex in presence of 100 mM KCl (Fig 7B). Though change in CD spectra in presence and absence of KCl is not very significant, the 265 nm positive peak and near 240 nm negative peak of the spectra suggest predominant formation of parallel G-quadruplex species. To delineate the precise guanines involved in structure formation in case of KD52 we carried out DMS assay in presence of 100 mM KCl. Along with no salt reaction, LiCl, known not to stabilize G-quadruplex, was used as a control. Results (Fig 7C, left panel) show protection of specific guanines with respect to LiCl reaction and the relative protection is quantified (Fig 7C, right panel). In addition to the two G stretches, G27, G25 and G24 of the GNG motifs at 3’ end showed protection towards DMS. Based on the sequence, CD, gel shift and DMS results, we built a 2-D model for potential intramolecular structure formation (Fig 7D). The model represents three G4 planes formed using two G stretches and two GNG motifs. Three bulges are also accommodated in order to form intramolecular G-quadruplex species (Fig 7D).

### Nucleolin and BG4 bind to G-quadruplex structures formed at SHOX gene using GNG motifs

**Fig 8:**
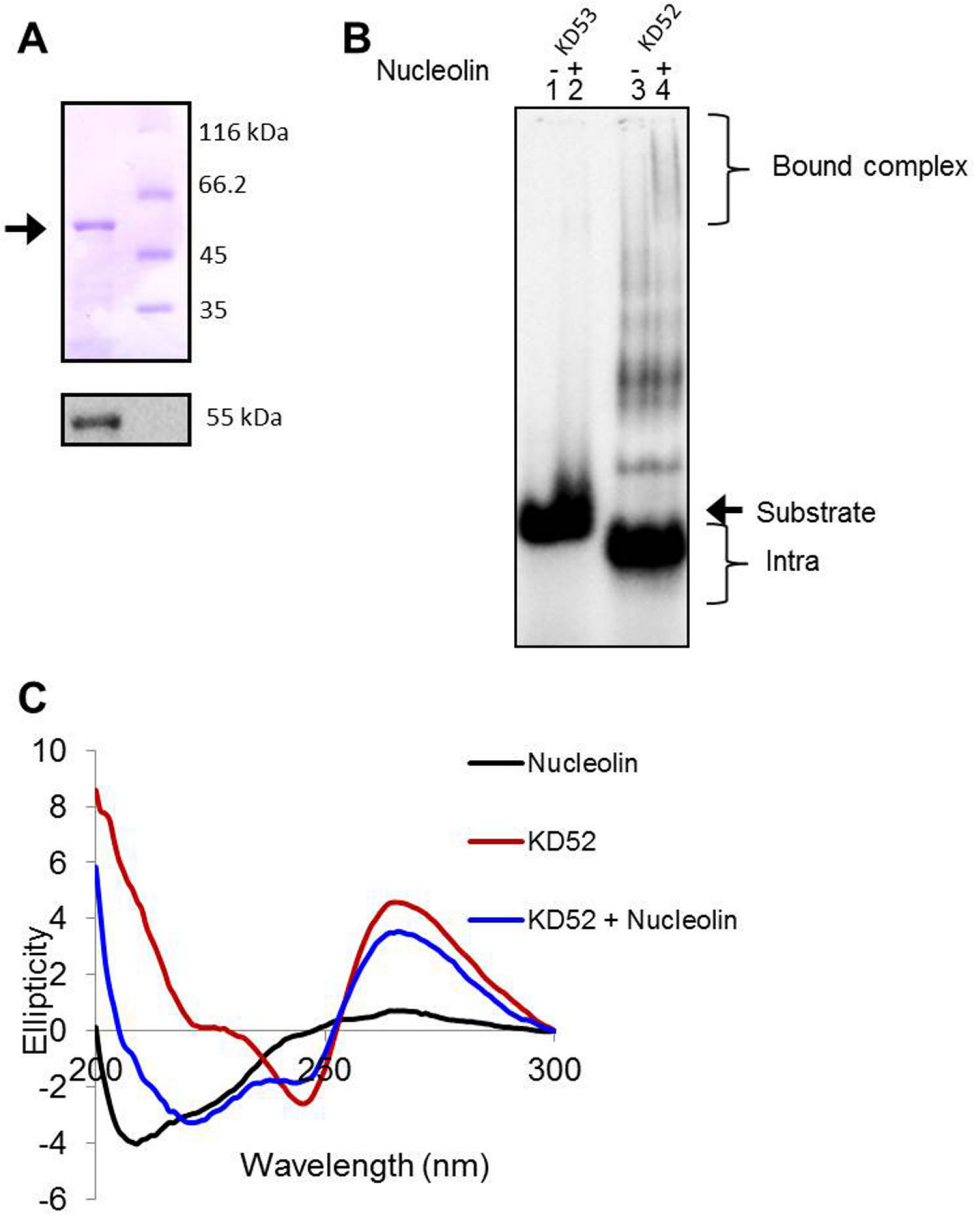
Binding of Nucleolin to G-quartets with GNGs as a structural component. (A) SDS PAGE profile showing purified recombinant His-tagged Nucleolin (55 kDa) (upper panel) and corresponding western blot using anti-His (lower panel). (B) Binding of purified recombinant Nucleolin to oligomers KD52 (harbouring two G stretches and GNG motifs) and its reverse complement KD53 in the presence of KCl (100 mM). (C) Analysis of CD spectra of recombinant His-tagged Nucleolin (20 μg) and its ability to bind KD52.

Nucleolin is an abundant eukaryotic nucleolar protein. It is well known in literature that Nucleolin binds G4 DNA efficiently. Some important examples include binding of Nucleolin to *c-MYC* and *VEGF* promoter G-quadruplexes [37–39]. Since in the previous section we found that two G stretches along with GNGs are sufficient to form G-quardruplexes, we envisaged binding of Nucleolin to these deviant structures. To test this, we purified recombinant His-tagged Nucleolin (55kDa), and confirmed its identity through western blotting (Fig 8A). Recombinant Nucleolin was then subjected to binding assays with KD52 in the presence of KCl (Fig 8B). Results showed specific binding of Nucleolin to KD52; whereas reverse complement oligomer, KD53 and a scrambled control, BTM6 were unable to bind to the Nucleolin (Fig 8B, lane 4; S5A and S5B Fig). This confirms the formation of the G-quadruplex structure utilizing two GNG motifs on KD52. Further, we performed CD analysis to evaluate the binding of Nucleolin to KD52 (Fig 8C). Interestingly, we observed a spectral shift in secondary structure of Nucleolin confirming its binding to parallel G-quadruplex formed on KD52.

**Fig 9:**
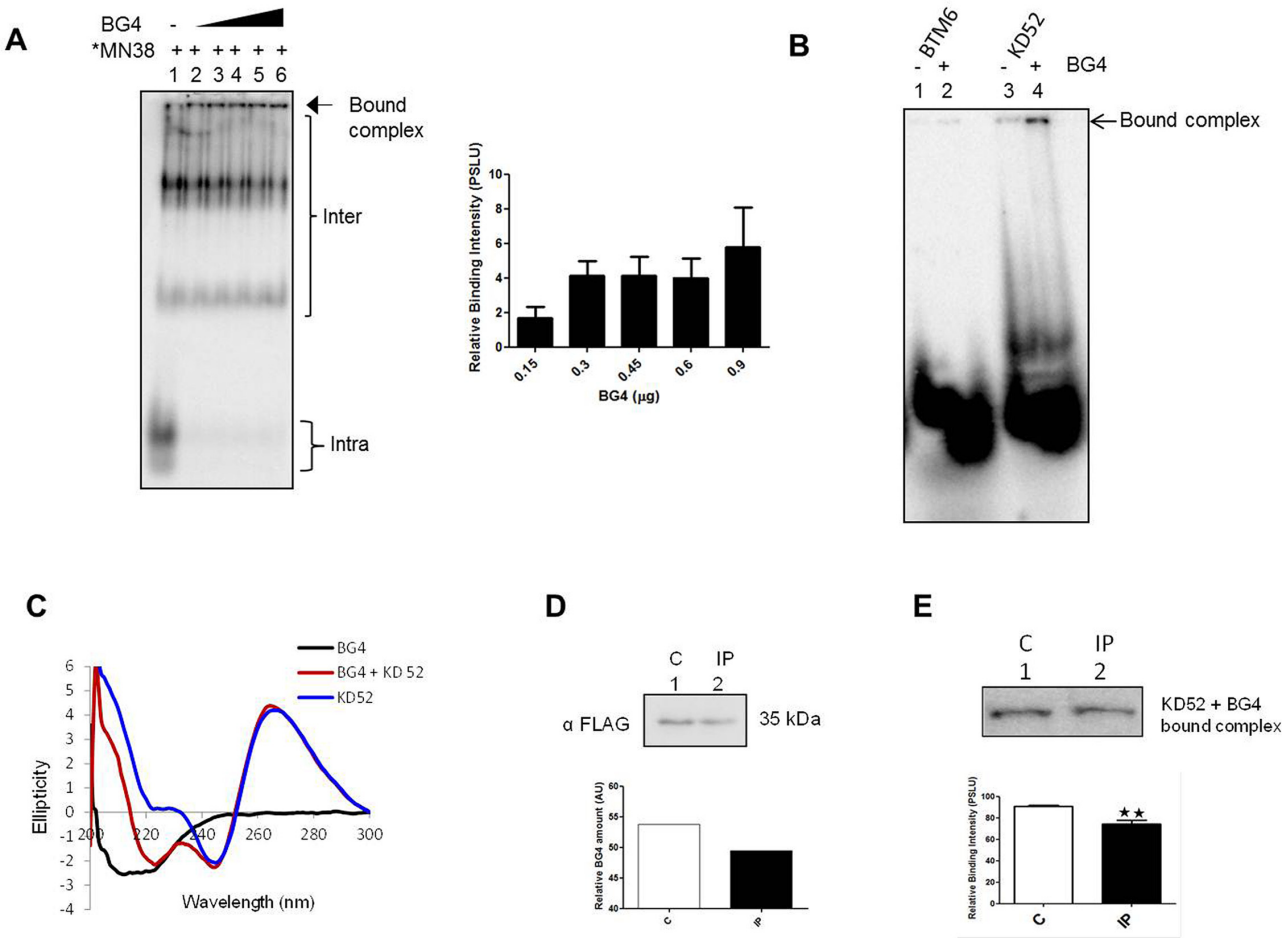
Evaluation of BG4 binding to G-quartets with GNGs as a structural component. (A) Binding of MN38 to increasing concentrations of BG4 (0.15, 0.3, 0.45, 0.6 and 0.9 μg; Lanes2-6) in presence of KCl (100 mM). (B) Binding of KD52 (G-quadruplex forming *SHOX* sequence) and a scrambled DNA control, BTM6 to BG4 (0.45 0078g) in presence of 100 mM KCl. (C) CD spectra of FLAG-tagged BG4 (5 μg) and its ability to bind KD52. (D) Immunoblot for BG4 (control and after IP) using anti-FLAG antibody. Quantification depicts relative reduction in BG4 level after IP. (E) Binding of BG4 to KD52. Upper panel depicts a representative gel image (6% native PAGE) for the relative binding of BG4 (between control and IP reaction) to KD52. Quantification based on multiple repeats is also shown.

BG4, an engineered, G-quadruplex structure specific antibody has been described previously [26]. It has been shown that BG4 can be used for probing G-quadruplexes with high selectivity and can be visualized on genomic DNA in human cells. We wondered whether G-quadruplexes with bulges can be recognized by BG4 antibody. To investigate this, we purified BG4 and analysed its binding to MN38 using EMSA in presence of KCl (Fig 9A). Robust binding of BG4 to intramolecular G-quadruplex species of MN38 involving both GNG and non-GNG was observed. However, the bound complexes could not be resolved beyond the wells possibly due to involvement of multiple antibody molecules in complex formation. Next we used KD52 and accessed its binding to BG4. In contrast to a random control BTM6, BG4 specifically bound to G-quadruplex forming DNA, KD52 (Fig 9B, lane 4). Circular dichroism analysis of KD52 in presence of BG4 showed a spectral shift suggesting antibody binding. Such shift was not observed when a control oligomer, KD53, which cannot form G-quadruplex was used (Fig 9C; S6 Fig). Further, to evaluate the specificity of BG4 binding, we carried out immunoprecipitation using anti-FLAG antibody (Fig 9D). The control and the IP reactions were analysed for immunodepletion through western blotting (Fig 9D). The control and IP supernatants were used for binding experiment with KD52. The results showed significant binding between BG4 and KD52 in case of control while the binding was reduced significantly following IP, reinstating the specificity of BG4 to KD52 (Fig 9E). Overall, our results provide novel insights into the role of GNG motifs in the formation of G-quadruplexes.

## Discussion

There are a variety of parameters that govern G-quadruplex structure formation. While most of the bioinformatics analyses done in search of putative G-quadruplex motifs use algorithms based on the conventional empirical formulae [9,40], increasing evidences have shown a lot of deviations from established conventions during G-quadruplex formation. Number of G-tetrads and loops are the major domains of deviations previously studied.

The human *c-KIT* promoter G-quadruplex forms a unique structure with a broken strand [41,42]. Although the sequence of this region harbours four, three guanine tracts, one of the G tetrad core is interrupted. This parallel three G tetrad structure involves two 1-nt strand reversal loops that connect two adjacent parallel stranded motifs. In another case, promoter element of human *c-MYB* gene contains three repeats of (GGA)_4_. This sequence majorly folds into a dimer which is composed of two parallel stranded unimolecular heptad: tetrad structures that involve two consecutive 5’ end (GGAGGAGGAGG) repeats [43].

Besides these unique deviations, the loop lengths of 1 nt to 15 nt have been shown to form stable G-quadruplex structures unlike the conventional 7 nt loop region. Studies have also shown that G-quadruplexes having two very short loops, could possibly accommodate one large loop [44]. Recently, in an elegant study, Phan’s group showed that large loops in G-quadruplex sequences can be included, only if these loops fold into duplex hairpins [28,29]. Thus, it appears that loops are no more restrictive between G-tracts, rather they can exist within the G tracts even when the length is more than 7 nt. These intervening loops, also referred as bulges, can be present at a variety of positions and can be composed of any nucleotide without affecting the stability. However, the length of the bulges could affect the stability considerably. A recent study also showed prevalence of previously uncharacterized noncanonical G-quadruplexes with bulges and long loops in the human genome using high throughput G4-seq [45].

In this context, our study for the first time shows that the involvement of GNG motifs in G-quadruplex structure formation is dependent on sequence and context. We have shown that among three genomic regions harbouring GNG motifs studied, only in two cases GNG motifs supported intramolecular G-quadruplex formation, when one of the G-stretches was mutated. Using *HOX11* G-quadruplex forming region as a model system, we have provided insights about the importance of stretches of guanines and how the choice of GNG utilization is altered with respect to the change in core sequences. Additionally, guanines in the loops, tracts and as GNGs, can compete towards formation of the G-tetrad, generating a variety of conformations. Importantly, it appears that even the length of GNG tract (GNG/GNGNG) may influence the G-quadruplex formation. Intriguingly, we observed that even two stretches of guanines may be sufficient for G-quadruplex formation, in presence of adjacent GNG motifs at *SHOX* gene, and can bind to well-studied G-quadruplex binding proteins Nucleolin [37,38] and recently discovered G- quartet antibody, BG4 [26]. It is important to note that we observed higher mobility of DNA substrates in mutants with respect to their respective wild type sequences in presence of KCl (Figs 1, 3, 4 and 6). As described before, this may be due to formation of G-triplexes [36].

Human genome is strewn with G rich sequences, which can fold into G-quadruplexes. Our study further elucidates the heterogeneity in accommodating GNG motifs during G-quadruplex formation although we are unable to draw definitive rules associated to usage of GNGs during structure formation. Importantly, our results support the probability of G-quadruplex formation using GNG motifs physiologically but in a context dependent manner. Whether all these motifs can fold into structure *in vivo* in a spatiotemporal fashion is yet to be answered. Telomere and promoter G-quadruplexes have been well characterized and their physiological role has been established [46]. These G-quadruplexes mostly follow the conventional empirical formula and are known to bind to different proteins exhibiting their physiological function.

As shown previously by us [21] and others, presence of G-quadruplexes may render a genomic region fragile owing to single-strandedness, which may eventually lead to genomic instability. G-quadruplex structure at the *BCL2* major breakpoint region is one of first studies to explain the fragility of the gene during t(14;18) translocation in Follicular Lymphoma [32,33,47,48]. Thus, increase in probability of G-quadruplex formation *in vivo* can have a huge impact both physiologically and pathologically. It is intriguing whether the GNG motifs can confer some additional specificity or function to these structures. Moreover, the nucleotides in the bulges along with those in loops may act as customised molecular scaffolds for proteins. Overall, more investigation is needed to understand the formation and role of G-quadruplexes using “GNG” sequences *in vivo*.

## Supporting Information

S1 Fig Circular dichroism spectra taken for GNG containing oligonucleotides. CD spectra for MN38, MS124, MS112, MS113 and MS114 in absence and presence of KCl (100 mM).
(JPG)

S2 Fig Assessment of role of 3^rd^ and 4^th^ stretches of guanines in G-quadruplex formation in the context of GNG motif. (A) The oligomeric sequence spanning region I of *HOX11* breakpoint region is shown. G-rich strand (MN38), its mutants (KD38, KD39, KD40, KD41) and complementary C rich strand (MN37) were designed and used for the study. Mutations were incorporated such that either the third G stretch or the fourth G stretch is altered to thymine (indicated in red) and keeping each as backbone the second GNG motif was mutated (indicated in red). (B) The G- and C-rich strands along with the mutants were incubated in the presence of KCl (100 mM) and resolved in the absence (left panel) or presence (right panel) of KCl (100 mM), in the gel and running buffer. For other details refer, Fig 1 legend. The grey boxed intra indicate intramolecular G-quadruplex species involving GNG motifs. Exposure increased part of gel image is indicated.
(JPG)

S3 Fig Circular dichroism spectra for GNG containing oligonucleotides. CD spectra for MS115 (A) and KD42 (B) in absence and presence of KCl (100 mM).
(JPG)

S4 Fig Refolding of intra molecular GNG G-quadruplexes after heat denaturation. Radiolabeled oligomeric DNA with G-rich, C-rich or mutant DNA sequences were heat denatured and gradually cooled and then resolved in the presence (right panel) of KCl (100 mM) in the gel and running buffer. (A) Native EMSA for KD53 and KD52 (*SHOX* oligomers, refer Fig 7A). (B) Native EMSA for MN89, RT17, KD47 and KD48 (*HIF1* alpha oligomers, refer Fig 6A, B). The substrate, intramolecular (Intra), and intermolecular (Inter) quadruplex structures are indicated. The red boxed ‘Intra’ indicate intramolecular G-quadruplex species involving GNG motifs.
(JPG)

S5 Fig Evaluation of binding of purified recombinant Nucleolin to oligomer, KD52. (A) EMSA study to analyse binding of Nucleolin to KD52 (harbouring two G stretches and GNG motifs) and a scrambled control BTM6 in the presence of KCl (100 mM). (B) Binding studies using increasing concentration of Nucleolin (6, 12, 24, 60 ng) to KD52.
(JPG)

S6 Fig Comparison of CD spectra of purified BG4 with KD52 and KD53. CD spectra of BG4 in presence of KD52 and KD53 (control) (shown in blue and red respectively).
(JPG)

S1 Table Sequence of oligonucleotides used in the study. (JPG)
