# Supplementary figures and images for: GNG Motifs Can Replace a GGG Stretch during G-Quadruplex Formation in a Context Dependent Manner
Source: PLoS One. 2016 Jul 14;11(7):e0158794. doi: 10.1371/journal.pone.0158794 (PMC4945072; doi:10.1371/journal.pone.0158794)

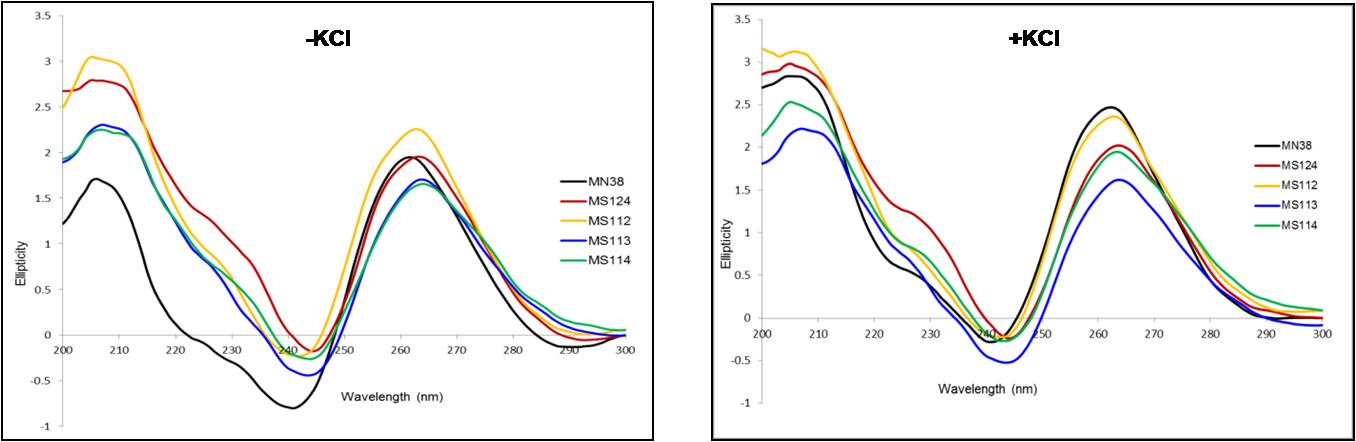

Supplement: S1 Fig — CD spectra for MN38, MS124, MS112, MS113 and MS114 in absence and presence of KCl (100 mM). (JPG) [file pone.0158794.s001.jpg]

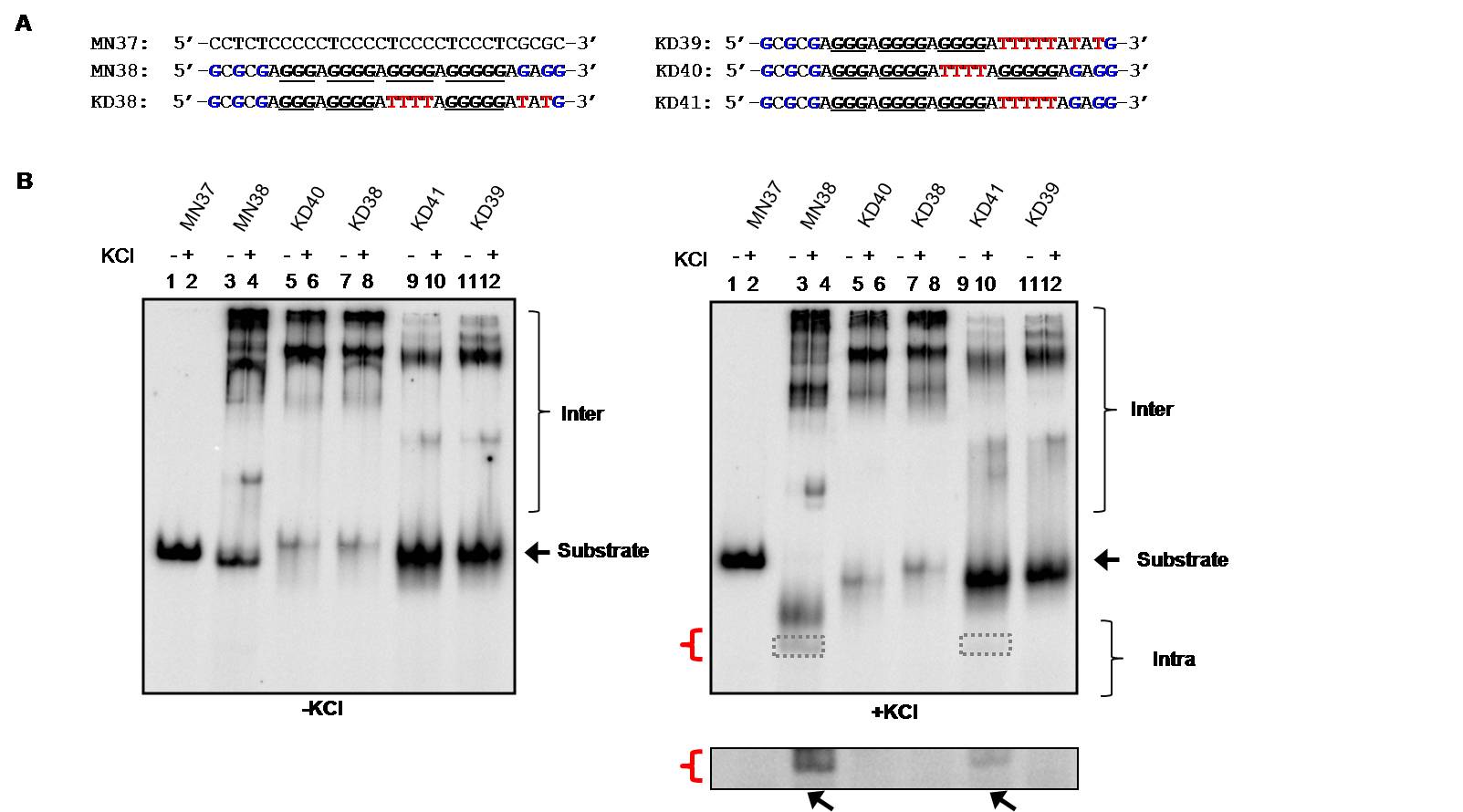

Supplement: S2 Fig — (A) The oligomeric sequence spanning region I of HOX11 breakpoint region is shown. G-rich strand (MN38), its mutants (KD38, KD39, KD40, KD41) and complementary C rich strand (MN37) were designed and used for the study. Mutations were incorporated such that either the third G stretch or the fourth G stretch is altered to thymine (indicated in red) and keeping each as backbone the second GNG motif was mutated (indicated in red). (B) The G- and C-rich strands along with the mutants were incubated in the presence of KCl (100 mM) and resolved in the absence (left panel) or presence (right panel) of KCl (100 mM), in the gel and running buffer. For other details refer, Fig 1 legend. The grey boxed intra indicate intramolecular G-quadruplex species involving GNG motifs. Exposure increased part of gel image is indicated. (JPG) [file pone.0158794.s002.jpg]

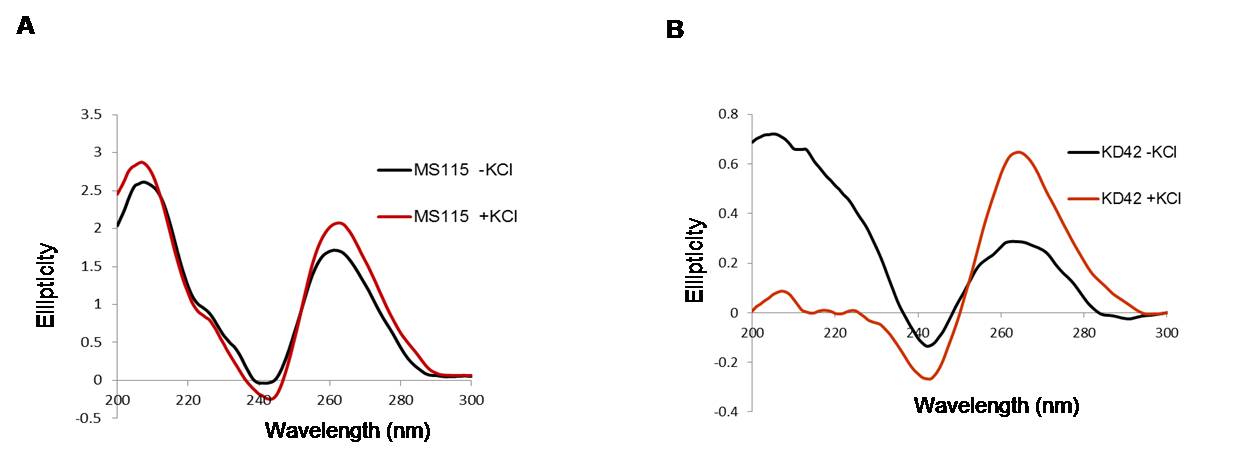

Supplement: S3 Fig — CD spectra for MS115 (A) and KD42 (B) in absence and presence of KCl (100 mM). (JPG) [file pone.0158794.s003.jpg]

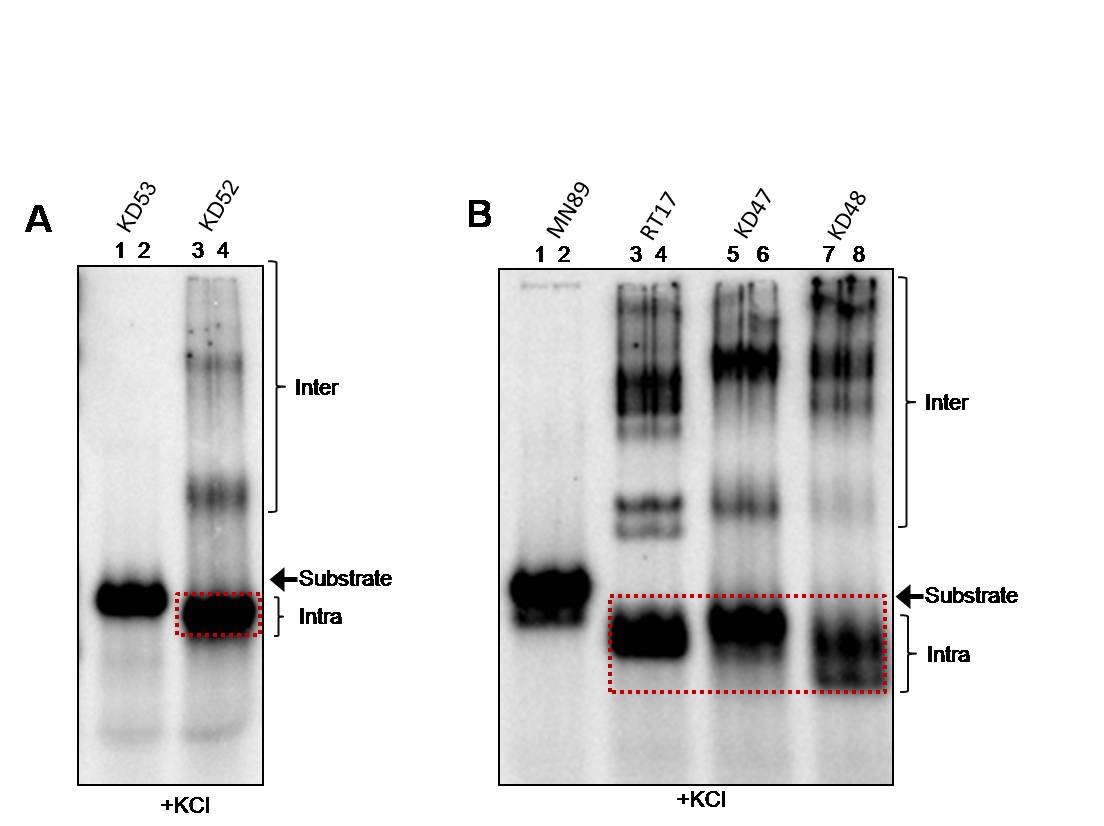

Supplement: S4 Fig — Radiolabeled oligomeric DNA with G-rich, C-rich or mutant DNA sequences were heat denatured and gradually cooled and then resolved in the presence (right panel) of KCl (100 mM) in the gel and running buffer. (A) Native EMSA for KD53 and KD52 (SHOX oligomers, refer Fig 7A). (B) Native EMSA for MN89, RT17, KD47 and KD48 (HIF1 alpha oligomers, refer Fig 6A, B). The substrate, intramolecular (Intra), and intermolecular (Inter) quadruplex structures are indicated. The red boxed ‘Intra’ indicate intramolecular G-quadruplex species involving GNG motifs. (JPG) [file pone.0158794.s004.jpg]

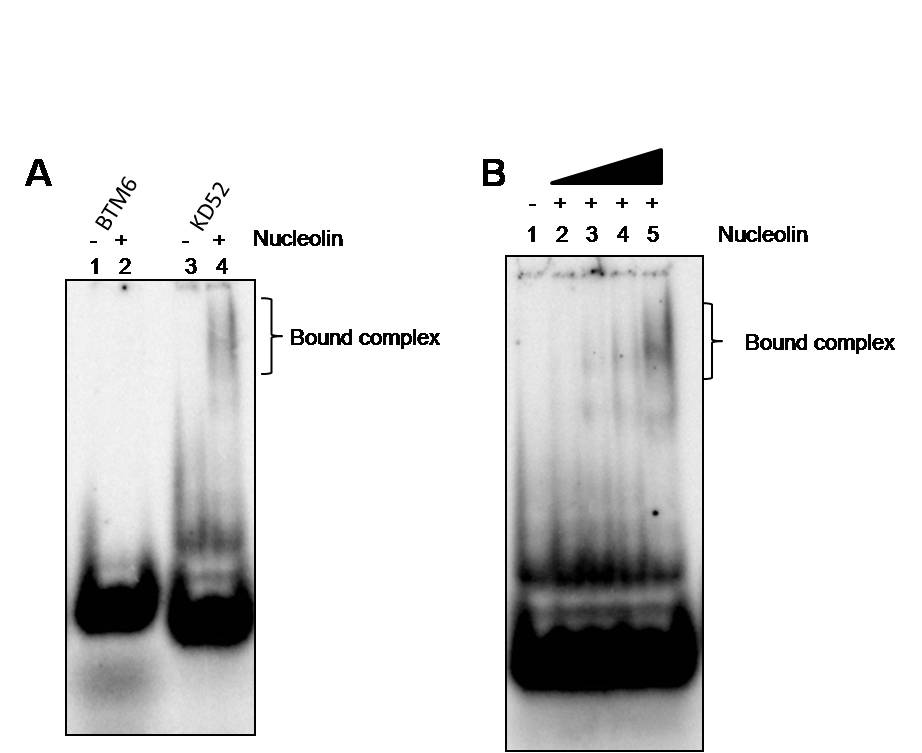

Supplement: S5 Fig — (A) EMSA study to analyse binding of Nucleolin to KD52 (harbouring two G stretches and GNG motifs) and a scrambled control BTM6 in the presence of KCl (100 mM). (B) Binding studies using increasing concentration of Nucleolin (6, 12, 24, 60 ng) to KD52. (JPG) [file pone.0158794.s005.jpg]

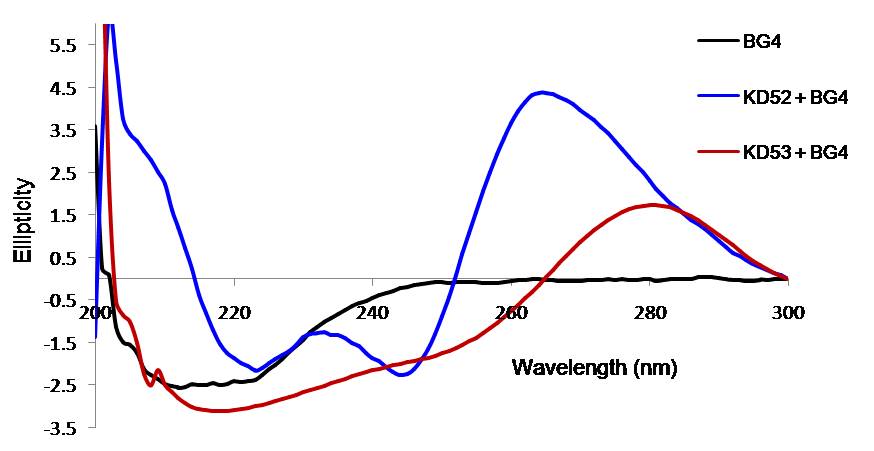

Supplement: S6 Fig — CD spectra of BG4 in presence of KD52 and KD53 (control) (shown in blue and red respectively). (JPG) [file pone.0158794.s006.jpg]

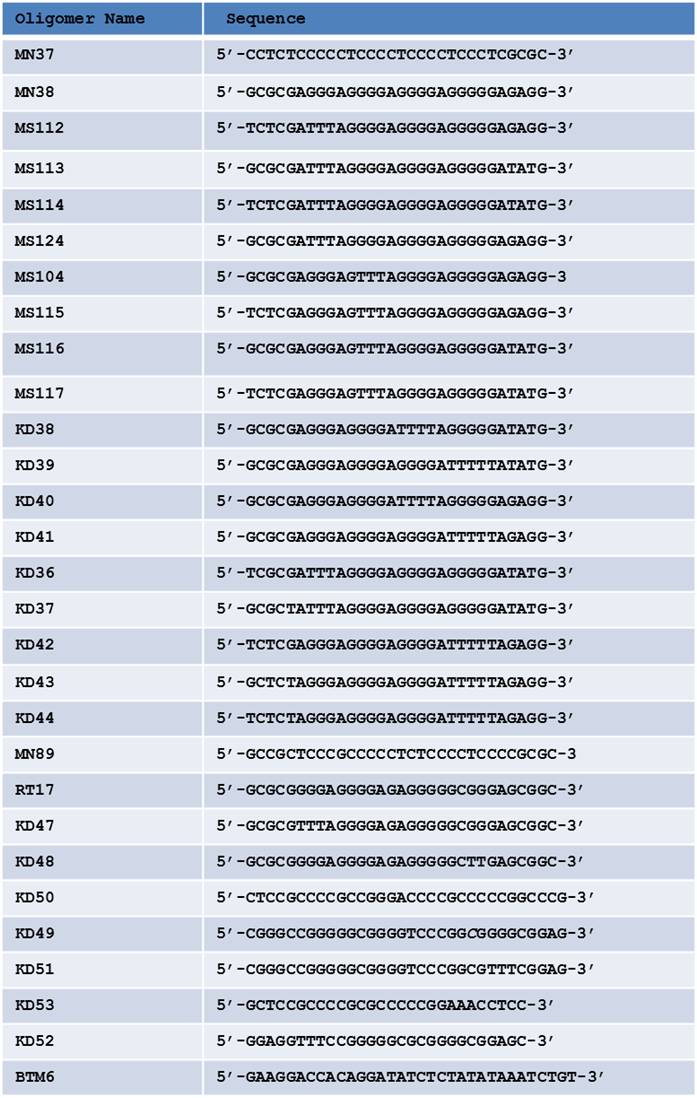

Supplement: S1 Table — (JPG) [file pone.0158794.s007.jpg]
